# From biomineralization to tumorogenesis—the expanding insight of the physiological and pathological roles of Fam20C

**DOI:** 10.1042/BSR20210040

**Published:** 2021-05-20

**Authors:** Yanbing Wu, Haoru Wang, Chao Liu

**Affiliations:** 1 Department of Stomatology, The Zhongshan College Affiliated to Dalian Medical University, Dalian, China 116085; 2 Department of Oral Pathology, School of Stomatology, Dalian Medical University, Dalian, China 116044

**Keywords:** cancer prognosis, Fam20C, microenvironment, phosphoproteome, phosphorylation, tumorogenesis

## Abstract

Fam20C is a Golgi kinase phosphorylating the majority of the secreted proteins. In this decade, the function of Fam20C has been largely disclosed in the loss-of function models. How the influence of the overexpressed Fam20C on cells or organs, and whether Fam20C was associated with tumorogensis still remain unknown. In the latest article in *Bioscience Reports*, a group from The Second Affiliated Hospital of Harbin Medical University established a correlation between the elevated Fam20C expression and the poor prognosis of multiple cancers (*Biosci. Rep.* (2021), **41**(1) BSR20201920). In addition, they also proposed the potential mechanisms how the increased Fam20C expression played a detrimental role in tumor progression by suggesting that the up-regulated Fam20C level affected the infiltration of immune cells and the capability of cancer metastasis. To give an overview of the expanding knowledge of Fam20C involved in the physiological and pathological events, we first reviewed the history of Fam20C study in this commentary, then, evaluated the correlation of the elevated Fam20C expression to the prognosis of multiple cancers, and finally, interpreted the perspectives that the Fam20C gain-of-function model was also critical for cancer therapy.

Kinase-catalyzed phosphorylation is a fundamentally post-translational modification key to protein configuration, activity, polymerization, etc. Hundreds of kinases have been identified for the phosphorylation of intracellular proteins, which play critical roles in the signal transduction, enzyme activity, protein assembly and disassembly. On the other hand, since the first secreted phosphoprotein (casein) was detected in 1883 [1], the phosphorylation of the extracellular proteins has been known more than one century. However, the kinase phosphorylating the extracellular or secreted proteins remains unknown until 2012, when this puzzle was deciphered by Dixon and his colleagues through the report that Family with sequence similarity 20 member C (Fam20C) could specifically phosphorylate the Ser-X-Glu/pSer motif in SIBLING proteins (small integrin-binding ligand, N-linked glycoprotein), the extracellular components essential for the mineralization of tooth and bone [2].

## The Fam20C-catalyzed phosphorylation in biomineralization

Family with sequence similarity 20 (Fam20) includes three members in mammals, Fam20A, Fam20B and Fam20C, all of which are homologous to the four-jointed Golgi protein kinases in *Drosophila* [1]. Fam20A was first detected in hematopoietic cells and reported as a pseudokinase facilitating the activity of Fam20C [3]. Fam20B was identified as a xylose kinase essential for the synthesis of glycosaminoglycan chains in proteoglycans [4]. Differing from FAm20A and Fam20B, Fam20C was proven to act as the kinase phosphorylating the secreted or extracellular proteins, and suggested to potentially influence the biomineralization and the extracellular microenvironment. However, because of the robust expression in ameloblasts, odontoblasts, cementoblasts and osteoblasts that would undergo mineralization [5–7], Fam20C was once named after dentin matrix protein 4 (DMP4) and thought as a matrix protein when it was first identified in mammals [8].

Although Fam20C was not considered as a kinase located in Golgi apparatus at the beginning, genetic screen demonstrated that Fam20C was the causative gene for Raine’s syndrome, a rare lethal osteosclerosis bone dysplasia in humans reported at 1989 [5,9]. Previously, Raine’s syndrome was characterized by the osteosclerosis in calvarium, ribs and long bones, the mid-face deformity, and the ectopic mineralization in brain and soft tissues, which made newborns died of aponea in several hours to days after birth [10]. Interestingly, taking the advantages of the genome-wide association study (GWAS) and the second generation sequencing technology from 2009, more and more patients arranging from the infants to the 80-year-old man were also found carrying Fam20C mutations [11]. Worthy of noticing, these subjects were predominantly suffering from hypophosphatemia rickets, with or without the sporadically ectopic over-mineralization in skeleton and soft tissues. Thus, the mutations in Fam20C confused the researchers until now with the the opposite manifestations. At the beginning, it was putatively thought that the different mutations differentially altered the kinase activity of Fam20C. Namely, the Fam20C mutations found in the osteosclerosis cases enhanced the kinase activity, while the mutations in the rickets patients suppressed the kinase activity. However, our unpublished studies showed that the transgenic mice only expressing the mutated Fam20C found in the lethal osteosclerosis cases gave rise to the similar phenotype to that of the transgenic mice whose Fam20C was replaced by the mutated Fam20C in the rickets cases. Both kinds of transgenic mice recapitulated the hypophosphatemia rickets as the Fam20C knockout mice did [12]. The conflict between the assumption and the expremental findings proposed a hypothesis that the loss of function of Fam20C primarily led to the hypophosphatemia rickets; however, some unknown environmental factors encountered by different individuals led to osteosclerosis in random locus. At all events, the concept of Raine’s syndrome is extended because of the novel manifestations found in the Fam20C-deficient survivals even though the underlying mechanisms are still unveiled. Worthy of noticing, the environmental clues mentioned above mainly refer to the microenvironment regulated by the phosphorylated targets of Fam20C.

The recent advances on the phosphoproteome of Fam20C supported the hypothesis that the microenvironment might contribute to the opposite manifestations of Raine’s syndrome. Our previous studies indicated a repressed BMP/Smad4 signaling in the Fam20C-deficient osteoblast and dental mesenchymal cell lines [13]. BMP2, 4 and 7 are speculated as the substrates of Fam20C because they possess the Ser-X-Glu/pSer motifs. Although the phosphorylation of BMP ligands by Fam20C still requires more evidence, the phosphorylation of FGF23 by Fam20C has been confirmed as a typical example that how the kinase activity of Fam20C modified the circulatory environment [14]. As an endocrine factor regulating serum phosphate concentration, FGF23 binds Klotho, the receptor in the tubular endothelium of kidney to promote phosphate excretion. Normally, Fam20C phosphorylates the Ser180 within the Arg176-His-Thr-Arg179/Ser180 motif in FGF23 to prevent Thr178 from the O-glycosylation, which makes the FGF23 accessible to furin for proteolysis. In the absence of Fam20C, because of the lack of the Ser180 phosphorylation, the Thr178 in FGF23 will be glycosylated, and the glycoaminoglycan chain will cover the cleave site between Arg179-Ser180. Thus, the uncleaved FGF23 will be sustained with a longer half-time in the circulation and promote the excretion of serum phosphate, resulting in the hypophosphatemia rickets. The recent discovery of Tagliabracci et al. enforced the notion that Fam20C could alter microenvironment by phosphorylating the secreted proteins [15]. In their study, more than two-thirds of the phosphoproteins in serum, plasma and cerebrospinal fluid (CSF) contained the Ser-X-Glu/pSer motif, and the phosphoproteome of Fam20C included, but was not limited to the signaling molecules, extracellular matrix and even the proteases. Therefore, Fam20C deficiency is strongly suggested to alter the extracellular environment by down-regulating not only the phosphorus concentration in the phosphoproteome but also the signaling and enzyme activity.

## The roles of Fam20C in tumorogenesis

Almost all the previous studies on Fam20C focused on its role in organogenesis, including the development of salivary glands [16], biomineralization [12,13], amelogenesis [17,18], etc., while few were involved in cancers. Recent studies have identified Fam20C as a key gene for the progression of lung adenocarcinoma in the context of hypoxia [19], and the Fam20C inhibitor, FL-1607 as an inducer for the apoptosis and an inhibitor for the migration of breast cancer cells [20,21]. Since the correlation between the increased Fam20C expression and the cancers was emerging, Liu et al. performed a comprehensive investigation by analyzing the data from the different database to testify the association of Fam20C with tumorogenesis [22]. Taking the advantage of the sufficient information on the subjects, they established the correlation not only between the Fam20C expression and the prognosis of multiple cancers, but also between the Fam20C expression and the extent of immune cell infiltration. Actually, their finding shed light on the future study how Fam20C worked in tumorgenesis.

To fill the gap between the physiological and pathological roles of Fam20C, Liu et al. analyzed Fam20C expression levels and the prognostic value in different types of cancers [22]. Combined the data from different databases, the Fam20C expression in cancers was found significantly elevated, particularly in central nervous system (CNS) cancers, breast invasive carcinoma (BRCA), cervical squamous cell carcinoma (CESC), colorectal cancer, esophageal carcinoma (ESCA), head and neck squamous cell carcinoma (HNSC), lymphoma, liver hepatocellular carcinoma (LIHC), lung cancer, pancreatic adenocarcinoma (PAAD), prostate adenocarcinoma (PRAD) and thyroid carcinoma (THCA). Compared with the abrogation or decreased Fam20C activity in Raine’s syndrome and hypophosphatemia rickets, the increased Fam20C expression detected in the variety of cancers implicated that the increased Fam20C activity was more detrimental to the cells. This finding is coincided with our unpublished experience that the transgenic mice for Fam20C overexpression died before weaning, whose phenotype is more severe than those in the Fam20C-deficient mice.

A latest study by Du et al. reported the secretary pathway kinase or kinase-like proteins signature for glioblastoma (GBM) prognosis [23]. In this report, they also found an evident correlation between the elevated Fam20C expression and the progressed glioma malignancy, as well as the coherence between the elevated Fam20C expression and the disrupted immune response in the GBM microenvironment. Similarly, the work of Li et al. in *Bioscience Reports* confirmed the widely and elevated expression of Fam20C among many cancer types, as well as its value for the detrimental prognosis in cancers, especially bladder urothelial carcinoma (BLCA), brain lower grade glioma (LGG) and stomach adenocarcinoma (STAD) [22]. Thus, the publications of both Du and Liu et al. indicated that the elevated Fam20C expression was associated with the detrimental prognosis, and the impaired immune cell infiltration in cancers. Although we know little about how the up-regulated Fam20C expression enhanced the progression or malignancy, it is implicated that the Fam20C expression level could be considered as a marker to predict the prognosis.

Tumor microenvironment (TME) plays a vital role in tumorogenesis. Acting as a critical component in TME of various cancer types, the infiltrating immune cells provide an accurate prognosis of tumors through their immune activities [24]. In the study by Liu et al., Spearman’s correlation coefficient via TIMER database was evaluated to correlate the Fam20C expression to the infiltration of immune cells in different cancers. They found that Fam20C expression was positively correlated with the infiltrating immune cells in TME, especially the neutrophils, CD4+ T cells, dendritic cells (DCs) and macrophages in BLCA, LGG and STAD. More evidently, by estimating the extent of correlation between Fam20C expression and infiltrating immune cells, the authors proposed the mechanisms through which Fam20C worked as a biomarker predicting the prognosis of cancers. They found that Fam20C potentially regulated tumor-associated macrophages (TAM) polarization by inducing Treg cell activation and T-cell exhaustion. Furthermore, a strong correlation between Fam20C expression and T helper cell markers (Th1, Th2, Th9, Tfh and Th17) in BLCA, LGG and STAD was also found. All these findings suggested that Fam20C played a role in tumorogenesis by regulating T-cell activation.

To further explore how Fam20C promotes the tumorogenesis. The authors also explored the correlation between Fam20C expression and the markers of epithelial–mesenchymal transition (EMT), including cadherin-2 (CDH2), E-cadherin (CDH1), snail, twist family bHLH transcription factor (TWIST), zinc finger E-box-binding protein 1 (ZEB1), and zinc finger E-box-binding protein 2 (ZEB2). Interestingly, the conversion of CDH1 to CDH2 was negatively correlated with Fam20C expression. In contrast, other EMT markers, including CDH2, SNAIL, TWIST, ZEB1 and ZEB2, were positively correlated with the elevated Fam20C expression. These data indicated that Fam20C could regulate EMT by phosphorylating cadherin-2 and other calcium-binding proteins, which were critical for cell adhesion, migration and invasion [15]. Coincidentally, the capabilities for cell adhesion, migration and invasion were essential for the lymph node metastasis in the cancers with the poor prognosis. Therefore, the elevated Fam20C expression most likely resulted in the poor prognosis in many cancers by regulating immune cell infiltration and cancer cell metastasis.

## Conclusion and perspectives

**Figure 1:**
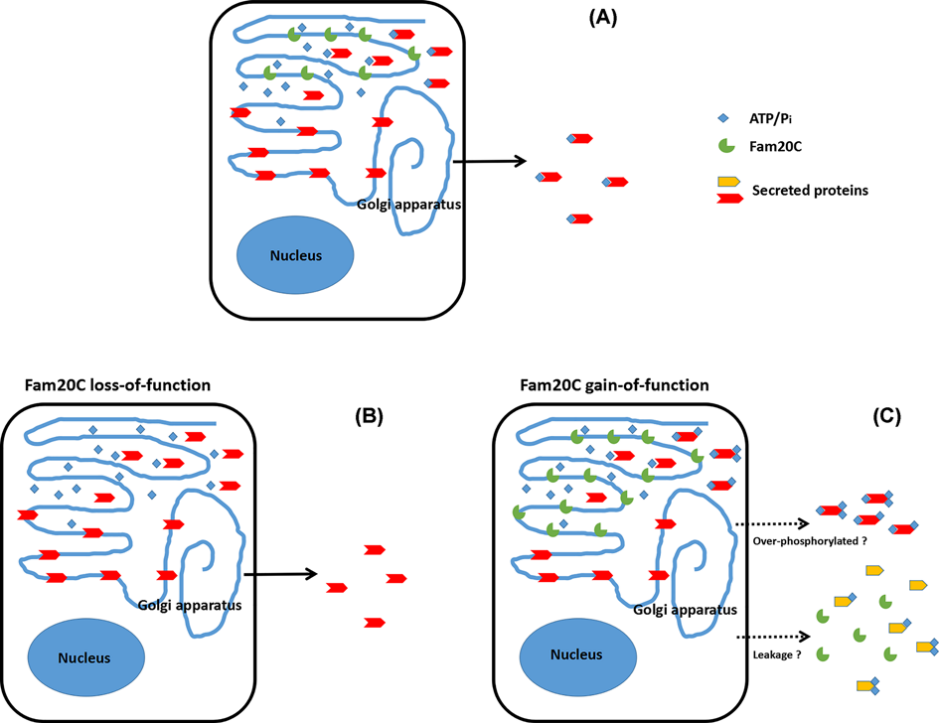
Perspective of the Fam20C loss- and gain-of-function studies (**A**) Phosphorylation of the secreted proteins by the normal expression of Fam20C. (**B**) In the absence of Fam20C, the lack of phosphorylation of the secreted proteins would alter the molecular configuration, enzyme activity and half-time. (**C**) The elevated Fam20C expression might result in the over-phosphorylation of the secreted proteins, which could change the microenvironment; whether the overexpressed Fam20C might be leaked into the extracellular space and phosphorylate the proteins in the extracellular space is still in debated.

In summary, the study of Liu et al. extended our understanding on FAM20C largely by testifying a putative concern whether Fam20C was associated with tumorogenesis. Since Fam20C is universally expressed in body and the substrates of Fam20C are widely distributed in the various body fluids and the extracellular space, it sounds reasonable that the alterations on Fam20C activity are capable of modifying the microenvironments. In the past decades, the contribution of the microenvironment to tumorogenesis has attracted lots of concerns [25,26], and the roles of extracellular signals in TME have also been documented [27–29]. The work by Liu et al. established the correlation between the elevated Fam20C expression and the deteriorating prognosis of multiple cancers by statistically analyzing the data from different databases. This work not only confirmed the prognostic value of increased Fam20C expression in cancers, but also suggested the mechanisms how the overexpressed Fam20C led to tumorogenesis. Actually, there are still a lot of known-unknown about Fam20C. For example, Fam20C phosphorylates the secreted proteins in Golgi apparatus; however, does Fam20C also phosphorylates the intracellular proteins? Or Fam20C could also be secreted into extracellular space and then, phosphorylate other secreted proteins in the extracellular space as the VTK does? [7,30] (Figure 1). All these answers will facilitate our understanding how the increased Fam20C results in the poor prognosis of multiple cancers. In the present study, although there is a lack of the biochemical, cellular and molecular proofs, the authors indeed provided the immune cells and EMT as the candidates for Fam20C mediated cancer progression and metastasis. More importantly, previous studies indicated that Fam20C could regulate the growth, adhesion and metastasis of cancer cells based on the Fam20C knock-down or knock-out models [15], but ignored the effects of overexpressed Fam20C on the physiological and pathological events (Figure 1). The present and the proceeding studies would most likely expand our knowledge of protein phosphorylation through the Fam20C gain-of function model. In practice, the enhanced Fam20C expression and the detrimental role in cancers make it possible as a biomarker to predict tumor prognosis. Potentially, the expanded insights of physiological and pathological functions of Fam20C will provide a novel strategy for the chemotherapy or immunotherapy of cancers by applying Fam20C as a new target.

## Abbreviations

BRCA: breast invasive carcinoma
CESC: cervical squamous cell carcinoma
CNS: central nervous system
DMP4: dentin matrix protein 4
ESCA: esophageal carcinoma
Fam20C: Family with sequence similarity 20 member C
GBM: glioblastoma
HNSC: head and neck squamous cell carcinoma
LIHC: liver hepatocellular carcinoma
PAAD: pancreatic adenocarcinoma
PRAD: prostate adenocarcinoma
THCA: thyroid carcinoma
TME: tumor microenvironment
